# Functional Involvement of Interferon-Inducible Transmembrane Proteins in Antiviral Immunity

**DOI:** 10.3389/fmicb.2019.01097

**Published:** 2019-05-16

**Authors:** Yuan Liao, Mohsan Ullah Goraya, Xu Yuan, Baoge Zhang, Shih-Hsin Chiu, Ji-Long Chen

**Affiliations:** ^1^Key Laboratory of Fujian-Taiwan Animal Pathogen Biology, College of Animal Sciences, Fujian Agriculture and Forestry University, Fuzhou, China; ^2^CAS Key Laboratory of Pathogenic Microbiology and Immunology, Institute of Microbiology, Chinese Academy of Sciences, Beijing, China

**Keywords:** interferon-inducible transmembrane proteins, ISGs, viral infection, interferon, innate immunity

## Abstract

Interferons (IFNs) play crucial roles in host defense against viral infections by inducing the expression of numerous IFN-stimulated genes (ISGs) that can activate host antiviral immunity. Interferon-inducible transmembrane proteins (IFITMs), a family of small transmembrane proteins, are critical ISG products. Compelling evidence has implicated that IFITMs can establish an innate immune state to eliminate pathogens efficiently. IFITM proteins can impede broad-spectrum viral infection through various mechanisms. It is generally believed that IFITMs can block the viral entry by suppressing viral membrane fusion. However, some findings indicated that IFITMs might also inhibit viral gene expression and viral protein synthesis and thereby impair viral replication. IFITMs may incorporate into virions during viral assembly and thus reduce the infectivity of nascent virions. The precise inhibitory mechanism of IFITMs on viral infection and replication still requires further exploration. In this review, we highlight the recent findings regarding critical roles of IFITMs in host-virus interaction. We also discuss the molecular mechanisms underlying their functions in antiviral responses.

## Introduction

In recent years, extensive studies have explored the innate defense mechanisms and cellular proteins involved in immunity against the infection of pathogens (Ishikawa and Barber, 2008; Unterholzner et al., 2010; Maarouf et al., 2018). Host innate immune response is triggered through the recognition of pathogen-associated molecular patterns (PAMPs) by pathogen recognition receptors (PRRs) (Alexopoulou et al., 2001; Yoneyama et al., 2004; Kato et al., 2006). The innate immune responses include induction of type I and type III interferons (IFNs) and subsequent expression of interferon-stimulating genes (ISGs) (Pulit-Penaloza et al., 2012; Wei et al., 2014). These ISGs encode specific proteins with distinct antiviral functions such as inhibitions of viral entry, viral gene transcription, viral protein synthesis, and viral particle assembly and release (Smith et al., 2014;Kane et al., 2016; Rabbani et al., 2016).

It is well-known that ISGs are critical for innate immunity against infection caused by human immunodeficiency virus (HIV-1), influenza A virus (IAV), West Nile virus (WNV), dengue virus (DENV), etc (Brass et al., 2009; Schoggins and Rice, 2011). Considerable efforts have been made to investigate the antiviral response of the ISG proteins in viral infection and replication, and to determine the underlying mechanisms. Of them, Interferon-inducible transmembrane proteins (IFITMs) are intensely induced during viral infection and play a crucial role in virus restriction. Recently, IFITMs have been identified as key ISGs that interfere with viral endosomal membrane fusion and the infectivity of nascent virions (Brass et al., 2009; Huang et al., 2011; Lu et al., 2011). In this review, we summarized the biological characteristics of IFITM genes, the antiviral properties of IFITM proteins, and their antiviral mechanisms.

## The IFITM Protein Family

### IFITM Genes

**FIGURE 1:**
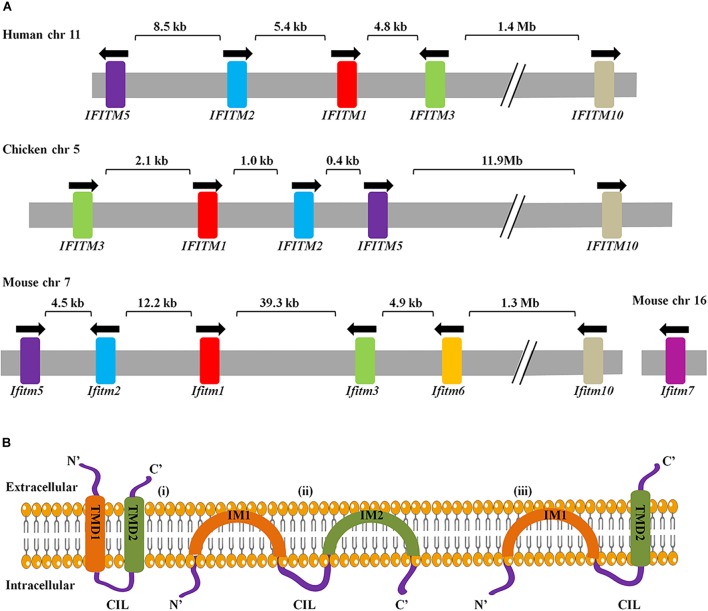
**(A)** Arrangement of IFITM genes cluster and genes topology. The arrangement of IFITM gene clusters in human, chicken, and mouse. Arrows indicate the direction of transcription. Exons are represented as color and introns are in gray. **(B)** Three topological models for IFITM proteins have been proposed. The first model represents the IFITM proteins as transmembrane molecules that have both the NTD and CTD extracellular with a CIL facing the cytoplasm. The second model represents IFITM proteins as intramembrane molecules where neither IM1 nor IM2 crosses the membrane and the NTD, CTD, and CIL all positioned intracellularly. Third, the most predominant models have an intracellular NTD and extracellular CTD.

In 1984, IFITM genes were first identified in interferon-treated TG98 neuroblastoma cells via cDNA screening with their transcripts named as 9-27, 1-8D, and 1-8U, also known as IFITM1, IFITM2, and IFITM3, respectively (Friedman et al., 1984). To date, IFITM1, IFITM2, IFITM3, IFITM5, and IFITM10 have been found in humans and their gene loci are located on chromosome 11. IFITM1, 2, 3, and 5 are clustered in a 26 kb region of the short arm, and IFITM10 is located 1.4 Mb apart. IFITM4P is a pseudogene in human. The mouse IFITM family consists of seven members, of which IFITM1, IFITM2, IFITM3, IFITM5, IFITM6, and IFITM10 are located on chromosome 7, while IFITM7 on chromosome 16. IFITM6 is located close to IFITM1, 2, 3, and 5 in mice but absent in humans (Sallman Almen et al., 2012). Homologous IFITM genes are also present in other species, including birds. Chicken IFITM genes are located on chromosome 5 (Siegrist et al., 2011; Hickford et al., 2012; Smith et al., 2013; Figure 1A).

### Subcellular Localization and Topology of IFITM Proteins

Several IFITM proteins are ubiquitously expressed in human primary tissues and cell lines. IFITM1 mostly concentrates on the lipid raft in the plasma membrane and early endosomes and interacts with some membrane proteins such as CD19 and CD81 (Smith et al., 2006; Weston et al., 2014). IFITM2 and IFITM3 are mainly in the intracellular compartments and colocalize with Rab7, CD63, lysosomal-associated membrane protein 1 (LAMP1) (Yount et al., 2012), and IFITM5 is primarily expressed in osteoblasts (Moffatt et al., 2008; Kasaai et al., 2013). IFITMs contain five domains, which consist of N-terminal domain (NTD), intramembrane domain (IMD), a conserved intracellular loop (CIL), transmembrane domain (TMD), and C-terminal domain (CTD) (Chen et al., 2017). The IMD and CIL comprise the CD225 domain, which is conserved in more than 300 proteins of the CD225/pfam 04505 family (John et al., 2013).

Interferon-inducible transmembrane proteins topology is key to understand how IFITM proteins can suppress virus infection by regulating membrane curvatures. There are three ideas for the topology of the IFITM proteins: first, both NTD and CTD localize extracellularly (Weidner et al., 2010); second, both NTD and CTD of IFTIM3 are located in the cytoplasm, rather than spanning out of the cellular membrane (Yount et al., 2012); the third and most recent model of IFITM3 explains that the NTD is present intracellularly and the CTD is spanning out of the cellular membrane (Bailey et al., 2013; Figure 1B). IFITMs topology varies among types of cells and stages of viral infection (Huang et al., 2011; Bailey et al., 2013). To date, IFITMs topology is not fully understood, and therefore needs further investigation.

### Biological Processes Involving IFITM Proteins

Interferon-inducible transmembrane proteins participate in various biological processes, such as immune response, germ cell homing and maturation, and bone mineralization. The IFITM family of vertebrates can be divided into three parts in phylogeny: immunity-related IFITM (IR-IFITM), IFITM5, and IFITM10 sub-families (Zhang et al., 2012). The IFITMs in clade I (IFITM1/2/3/6/7) are associated with innate immunity, and their expression can be induced by IFNs. IFITM5 and 10 undergo functional and adaptive evolution rather than positive selection (Moffatt et al., 2008; Hanagata et al., 2011; Bailey et al., 2014). In addition, oncostatin M and IL-6 can also induce the IFITM3 expression via JAK-STAT signaling pathway (Bailey et al., 2012), suggesting that expression of IFITM3 is not only dependent on IFNs but also modulated through various cellular factors.

Recent studies proved that IFITM proteins are associated with the transduction efficiency of lentiviral vector. Human and pig IFITM proteins partially limited the transduction of VSV-GFIV and GP64-FIV, thereby limiting the transfer of genes based on lentiviral vectors to airway epithelial cells (Hornick et al., 2016). It was observed that H37Rv-mCherry signal was weaker in IFITM3-overexpressing cell lines compared to cells transduced with empty lentiviral vector and IFITM1 and IFITM2 overexpression vector. Moreover, IFITM3 overexpression can significantly inhibit the growth of *Mycobacterium tuberculosis* in monocytes (Ranjbar et al., 2015), indicating its clinical potential for treatment of the disease caused by *Mycobacterium tuberculosis*.

## Antiviral Roles of IFITM Proteins and Their Underlying Mechanisms

Interferon-inducible transmembrane proteins have been characterized as critical cellular factors involved in immune response to a broad range of viruses (Table 1), including IAV (Feeley et al., 2011), HIV-1 (Lu et al., 2011), WNV, DENV (Jiang et al., 2010), vesicular stomatitis virus (VSV) (Weidner et al., 2010), SARS Coronavirus (SARS-CoV), and Marburg virus (MARV) (Huang et al., 2011). Palmitoylation of cysteine is required for the antiviral function of IFITMs (Yount et al., 2010). IFN-inducible IFITM proteins contain conserved cysteine residues which join the CIL and the putative membrane-interacting domains. Substituting cysteines with alanines reduces the clustering on the membrane and impairs the antiviral activity of IFITM3 (John et al., 2013). Non-ubiquitinated and S-palmitoylated IFITM3 is intracellular in nature and manifests potent antiviral activities (Yount et al., 2012).

At present, no clear consensus has been reached on the integrated antiviral mechanism of IFITMs, although a majority of researchers believe that IFITM proteins target viruses by preventing the virus-cell fusion. However, previous studies have uncovered that IFITMs restrict virus replication by regulating the viral protein expression and reducing the infectivity of nascent viruses (Compton et al., 2014; Tartour et al., 2014, 2017).

**Table 1:** List of RNA and DNA viruses restricted by IFITM proteins.

Family	Viruses	Envelop	pH dependency	References
**RNA viruses**				
*Orthomyxoviridae*	Influenza A and B viruses	Yes	^∗∗^	Brass et al., 2009; Smith et al., 2013
*Flaviviridae*	West Nile virus, Dengue virus, Hepatitis C virus, Avian tembusu virus, Zika virus	Yes	^∗^, ^∗∗^, ^∗∗^, ^∗∗^, ^∗∗^	Brass et al., 2009; Everitt et al., 2012
*Rhabdoviridae*	Vesicular stomatitis virus, Rabies virus, Lagos Bat virus	Yes	^∗^, ^∗∗^, ^∗∗^	Weidner et al., 2010; Smith et al., 2013
*Bunyaviridae*	La Crosse virus, Hantaan virus, Andes Virus, Rift valley fever	Yes	^∗∗^	Mudhasani et al., 2013
*Filoviridae*	Ebola virus, Marburg virus	Yes	^∗^	Huang et al., 2011
*Alphaviridae*	Sindbis and Semliki Forest Virus	Yes	^∗^	Weston et al., 2016
*Coronaviridae*	SARS Corona virus	Yes	^∗∗^	Huang et al., 2011
*Retroviridae*	HIV-1, Jaagsiekte sheep retrovirus (JSRV)	Yes	No, ^∗∗^	Brass et al., 2009; Li et al., 2013
*Reoviruses*	Reovirus	No	^∗∗^	Anafu et al., 2013
**DNA viruses**				
*Asfarviridae*	African swine fever virus	Yes	^∗∗^	Munoz-Moreno et al., 2016
*Poxviridae*	Vaccinia virus	Yes	^∗∗^	Li et al., 2018
*Iridoviridae*	Rana grylio virus	Yes	^∗∗^	Zhu et al., 2013


*^∗^Fusion at pH > 6, ^∗∗^Fusion at pH < 6.*

### IFITM Proteins Restrict Viral Entry Into Target Cells

Increasing evidence has shown that IFITMs may restrict viral entry by inhibiting fusion with plasma membrane and endosomal or lysosomal membranes (Brass et al., 2009; Bailey et al., 2014; Tartour et al., 2017). Recently, a vital endocytic signal (20-YEML-23) that can guide the endocytosis of IFITM3 has been identified (Jia et al., 2014). IFITM proteins are involved in the enzymatic activity of cathepsin L (Huang et al., 2011), which is essential for the fusion of some enveloped viruses with endosomes of host cells (Zhou et al., 2016). Therefore, manipulating IFITM proteins can impact the entry of some enveloped viruses. Furthermore, IFITM proteins modify the pH of endosomes or lysosomes by accumulating non-specific proteases, thereby altering the lipid concentration of vesicle membrane or the activity of V-type proton ATPase (Wee et al., 2012). Low endosomal pH changes the conformation of viral envelope proteins, such as hemagglutinin (HA) (Sieczkarski and Whittaker, 2005), leading to the hemifusion of viral membrane with endosomal membrane. At the optimum pH, IFITM2, and IFITM3 can mediate the inhibition of IAV by influencing the pattern and duration of virus co-localization with IFITM proteins (Gerlach et al., 2017). IFITM3 restricts the entry of enveloped viruses by preventing the hemifusion of viral particles with either plasma or endosomal membranes (Li et al., 2013). Moreover, some non-enveloped viruses, such as reovirus, can be restricted by IFITM3 through regulation of late endosome functions during cell entry (Anafu et al., 2013).

In addition, it has been revealed that IFITMs overexpression changes the physical properties of cellular membranes and inhibits the fusion of pore formation, but the functional explanations vary on these mechanisms. One theory is that IFITM3 interacts with vesicle-membrane-protein-associated protein A (VAPA) and disrupts its interaction with the oxysterol-binding protein (OSBP) that controls the cholesterol content of endosomal membranes. Through this mechanism, IFITM3 enriches cholesterol in the membranes of cellular compartments containing lysobisphosphatidic acid (LBPA) and CD63, resulting in reduced fluidity and increased rigidity of the membrane and thus decreasing viral fusion (Amini-Bavil-Olyaee et al., 2013). However, Desai et al. (2014) have found that other methods leading to cholesterol accumulation in late endosomes cannot inhibit viral fusion unless IFITM3 is overexpressed, suggesting that the mechanism by which IFITM3 inhibits viral fusion may not depend on the increase of cholesterol in late endosomes.

### IFITM Proteins Can Restrict Viral Assembly and Reduce Infectivity of Nascent Virions

Interferon-inducible transmembrane proteins potentially affect the fusion with intralumenal vesicles within multivesicular bodies/late endosomes and redirect viruses to a non-productive pathway. Overexpression of IFITM proteins enlarges the acidified compartments, suggesting that these proteins interfere with endosomal trafficking or fusion of vesicles carrying viral components (Feeley et al., 2011). However, down-regulation of IFITM proteins have no effect on acidified compartments size or restriction efficiency, regardless of increased IAV replication in cells (Brass et al., 2009; Huang et al., 2011).

A recent research has shown that the IFITM2 and IFITM3 may reduce the infectivity of viruses in two ways: regulating virus-endosome fusion rates and accelerating the trafficking of virus-endosome to lysosomes (Spence et al., 2019). Moreover, by constructing a functional IFITM3 tagged with fluorescent proteins, it has been observed that IAV can undergo hemifusion in the IFITM3-positive endosomes but fail to release viral components. Meanwhile, IFITM3 blocks viral fusion by accumulating in the endosomes containing IAV (Suddala et al., 2019). These findings suggest that IFITM proteins may limit viral infection by promoting transportation of viral particles into lysosomes.

Interferon-inducible transmembrane proteins can also reduce the infectivity of newly produced viruses along with the endosomal vesicle restriction (Tartour et al., 2014, 2017). For example, IFITM proteins colocalize with envelope glycoprotein (Env) and group-specific antigen (Gag) proteins of developing HIV-1 virions and subsequently become a part of nascent viral particles, thereby inhibiting the entry of nascent virions into new host cells (Compton et al., 2014; Tartour et al., 2014; Yu et al., 2015). However, not all viruses can be restricted by IFITM proteins. For instance, the infectivity of Rift Valley fever virus (RVFV), Mopeia virus (MOPV), and Adeno-associated virus (AAV) is not affected by IFITM proteins (Tartour et al., 2017).

### IFITM Proteins Can Inhibit Viral Protein Synthesis

Recently, a novel mechanism by which IFITM proteins restrict viral infection has been identified. It shows that IFITM suppresses HIV-1 protein synthesis by excluding viral mRNA transcripts from polysomes, which can be rescued through expression of the viral accessory protein Nef. The observation indicates that IFITM-mediated HIV-1 restriction takes place at the translational level (Lee et al., 2018).

## The Spectrum of IFITM-Restricted Viruses

Interferon-inducible transmembrane proteins suppress virus pathogenesis through three strategies: restricting viral entry into target cells (Brass et al., 2009); incorporating of IFITMs into virions during viral assembly and thus reducing viral infectivity (Tartour et al., 2017); inhibiting viral protein synthesis (Lee et al., 2018). It is well known that IFITM proteins can restrict RNA viruses. Recently, increasing evidence demonstrates that IFITMs can also restrict some DNA viruses (Munoz-Moreno et al., 2016; Li et al., 2018). However, IFITM proteins might not affect the pathogenesis of most DNA and non-enveloped viruses, although it was shown that IFITMs restricted non-enveloped reoviruses (Anafu et al., 2013). The antiviral activity of IFITMs depends on various factors, including viral titer, host cell type, and expression level of IFITM proteins.

### RNA Virus

#### Orthomyxoviridae

The antiviral activity of IFITMs (IFITM1, IFITM2, and IFITM3) against IAV is observed in a RNA interference screen for host factors (Brass et al., 2009; Chen et al., 2018). Depleting these IFITM proteins by small RNA interference enhances the replication of IAV, while overexpression of them reduces the virus replication. The restriction by IFITM proteins occurs at the early replication of IAV, and IFITM3 has a more pronounced effect than IFITM1 and IFITM2. *In vivo*, the absence of IFITM3 results in uncontrolled replication of H1N1 and H3N2 influenza A virus in the lungs and high morbidity of the infected animals (Bailey et al., 2012). VAPA and OSBP mediate intracellular cholesterol homeostasis to regulate virus release into the cytosol. The interaction between VAPA and OSBP can be disrupted by IFITM3, resulting in cholesterol accumulation in the late endosome and thereby suppression of the entry of IAV (Amini-Bavil-Olyaee et al., 2013). Amphotericin B can rescue IFITM3-induced IAV restriction by binding to sterol and causing membrane-spanning pore formation and ion egress (Lin et al., 2013). Another study indicates that IFITM3 may restrict IAV through blocking the formation of fusion pores at the post-hemifusion stage rather than accumulating excess cholesterol in the late endosome (Desai et al., 2014). Mice lacking IFITM3 alone are more susceptible to IAV and exhibit higher mortality and viral burden, and their phenotypes are similar to those lacking entire IFITM locus (Bailey et al., 2012). In humans, single nucleotide polymorphisms (SNPs) within the coding region of the IFITM3 gene can alter the antiviral response to IAV infection, such as SNP rs12252-C. SNP rs12252-C bears T/C substitution mutation to alter a splice acceptor site, which encodes a truncated form of IFITM3 lacking its N-terminal 21 amino acids and thereby leads to a compromised anti-IAV activity of IFITM3. Individuals with SNP rs12252-C/C homozygotes show more severe symptoms and higher mortality than heterozygotes following IAV infection (Wang Z. et al., 2014; Yang et al., 2015). Additionally, eukaryotic translation initiation factor 4B (eIF4B), which can be down-regulated by IAV NS1, is capable of modulating the expression of IFITM3 (Wang S. et al., 2014).

#### Flaviviridae

Several viruses of the Flaviviridae family, including DENV, yellow fever virus (YFV), WNV, Zika virus (ZIKV), and hepatitis C virus (HCV), have aroused global health concern. Numerous studies have demonstrated that IFITM proteins have the ability to restrain flavivirus infection (Brass et al., 2009; John et al., 2013; Savidis et al., 2016; Chen et al., 2017). IFITM1, IFITM2, and IFITM3 have been proved to restrict DENV, YFV, WNV, and Omsk hemorrhagic fever virus (OHFV) by blocking virus entry (Brass et al., 2009; Jiang et al., 2010). Knockout of IFITM3 in mice increased mortality of the animals upon subcutaneous infection with WNV (Gorman et al., 2016). The replication of ZIKV can be inhibited by both IFITM1 and IFTM3, but IFITM3 exerts a more effective inhibition than IFITM1, which occurs at the early stage after viral fusion prior to its early RNA transcription (Savidis et al., 2016). Moreover, overexpression of IFITM3 is able to prevent cytopathicity mediated by ZIKV, such as cell death (Monel et al., 2017). IFITM1 has also been found to disrupt the entry of HCV through interaction with viral coreceptors, CD81 and occludin, and inhibit the viral replication (Raychoudhuri et al., 2011; Bhanja Chowdhury et al., 2012; Wilkins et al., 2013). A recent study has shown that IFITM2 and IFITM3 can also limit the replication of HCV at the late stage of viral entry (Narayana et al., 2015).

#### Filoviridae and Coronaviridae

Interferon-inducible transmembrane proteins can also efficiently restrict filoviruses and coronaviruses. Viruses of these two families share a common late endocytic enzymatic system by which lysosomal cysteine protease cathepsin L mediates the proteolytic cleavage of fusion proteins to infect the target cells (Chandran et al., 2005; Huang et al., 2006). IFITM1, IFITM2, and IFITM3 are capable of restricting GP1, 2-mediated entry and subsequently MARV and Ebolavirus (EBOV) replication, and the entry of filoviruses is suppressed by treatment with IFN as well. Collectively, IFITMs and IFNs can inhibit virus replication by entry restriction (Huang et al., 2011). In comparison with IAV, filoviruses are more sensitive to IFITM1 and murine IFITM5 and IFITM6. SARS-CoV S protein-mediated entry is also restricted by IFITM1, IFITM2, and IFITM3. Recently, mutations within residues and structural motifs of IFITMs are found to modulate the entry of coronaviruses. For instance, substitution of Y20 in IFITM3 with either alanine or aspartic acid enhances SARS-CoV entry, and the IFITM3 Y99A or Y99D mutants exhibits a reduced activity against Middle East respiratory syndrome coronavirus (MERS-CoV) entry (Zhao et al., 2018).

#### Retroviridae

Interferon-inducible transmembrane proteins were thought to be unable to interfere with HIV-1 infection (Brass et al., 2009). However, subsequent investigations have shown that IFITM2 and IFITM3 can restrict HIV-1 entry (Lu et al., 2011; Chutiwitoonchai et al., 2013; Compton et al., 2016; Lee et al., 2018). Moreover, non-human primate IFITM proteins can also suppress HIV and Simian immunodeficiency virus (SIV) (Wilkins et al., 2016). The Env is a vital factor in promoting HIV-1 transmission (Ding et al., 2014; Wang et al., 2017). The structure of viral particles (e.g., capsid core) and Env affect the extent of IFITMs restriction joint (Wrensch et al., 2017). IFITM1, IFITM2, and IFITM3 colocalize with HIV-1 Gag and Env proteins and incorporate into nascent virions during assembly in virus-producing cells, which subsequently reduces the viral infectivity and inhibits virus fusion and spread (Compton et al., 2014; Tartour et al., 2014). Possibly, IFITM2 and IFITM3 interact with Env, impair its processing and restrict virus infection, but the IFITMs restriction can be overcome by Env mutants (Yu et al., 2015). HIV-1 requires engagement of its Env with primary receptor CD4 and a chemokine receptor CCR5 or CXCR4 to enter the target cells (Wu et al., 2017). CXCR4-tropic viruses are found more susceptible to IFITM2 and IFITM3, whereas IFITM1 preferentially restricts CCR5 counterpart, indicating that IFITMs differentially inhibit HIV-1 replication contingent on its coreceptor tropism (Foster et al., 2016). Together, these data reveal that IFITMs restrict HIV-1 infection and replication through inhibiting viral entry and viral gene expression.

#### Rhabdoviridae, Bunyaviridae, and Alphaviridae

Interferon-inducible transmembrane proteins can restrict the infection of other enveloped viruses, including rhabdoviruses, bunyaviruses, and alphaviruses (Alber and Staeheli, 1996; Mudhasani et al., 2013; Xu-Yang et al., 2016). The replication of VSV, a member of the *Rhabdoviridae* family, can be inhibited by human IFITM1 (Alber and Staeheli, 1996). Furthermore, IFITM3 can inhibit VSV glycoprotein-mediated pseudovirus entry and primary transcription of VSV genome. Both N-terminal 21 amino acid residues and C-terminal transmenbrane region of IFITM3 are functional in its antiviral activity (Weidner et al., 2010). Variable restriction of IFITMs on viruses has been exhibited in the *Bunyaviridae* family. IFITM2 and IFITM3 impede viral membrane fusion with endosomes to restrict RVFV infection. IFITM1, 2, and 3 all have a board-spectrum antiviral activity against several other bunyaviruses, including La Crosse virus (LACV), Hantaan virus (HTNV), and Andes virus (ANDV). In contrast, none of the IFITMs restricts the infection of Crimean-Congo hemorrhagic fever virus (CCHFV). The efficiency of cell-cell fusion mediated by Semliki Forest virus (SFV), an alphavirus, fusion protein E1/E2 is also markedly reduced by IFITM1 and IFITM3 (Li et al., 2013). In addition, IFITMs, especially IFITM3, can restrict SFV capsid release from endosomes and fusion at the plasma membrane (Weston et al., 2016). *In vivo*, IFITM3 KO mice have shown more severe clinical outcomes with higher levels of alphaviruses titer and increased proinflammatory cytokines in multiple organs (Poddar et al., 2016).

#### Reoviridae

Reovirus is the only non-enveloped virus whose infection and replication can be restricted by IFITM3 (Anafu et al., 2013). IFITM3 restricts the reovirus infection by regulating Rab7-containing late endosome functions via delaying virus entry and escape as well as the proteolysis of viral outer capsids. In consistence with this observation, IFITM3 expression does not alter the entry of reovirus into the infectious subvirion particles (ISVPs), so endosomal acidification is not required. Together, these results indicate that IFITM3 targets reoviruses at the level of endosomal penetration.

### DNA Viruses

Although IFITM proteins are known to restrict a board spectrum of RNA viruses, little is known about their antiviral effects on DNA viruses. PoIFITM1, the fish IFITM1 isolated from flounder *Paralichthys olivaceus*, blocks Rana grylio virus (RGV) replication at the viral entry stage (Zhu et al., 2013). However, the overexpression of IFITM1, IFITM2, and IFITM3 is unable to inhibit the infection of human papillomavirus (HPV), human cytomegalovirus (HCMV), and adenovirus type 5 (Ad5) although type I IFNs can efficiently reduce HPV infection. Conversely, IFITM1 and IFITM3 overexpression even enhances HPV infection (Warren et al., 2014). IFITM1 also enhances the infectivity of Kaposi’s sarcoma-associated herpesvirus (KSHV), Epstein-Barr virus (EBV), and herpes simplex virus type 2 (HSV-2) (Hussein and Akula, 2017). Recently, IFITM proteins are reported to impact the infectivity of African swine fever virus (ASFV) and the endocytosis-mediated entry of ASFV. Possibly, IFITM2 and IFITM3 inhibit the ASFV entry by altering the membrane fusion and cholesterol endosomal efflux (Munoz-Moreno et al., 2016). Moreover, overexpression of IFITM3 protein significantly restricts vaccinia virus (VACV) replication by limiting virus binding and interfering viral entry in a low pH-dependent fusion (Li et al., 2018).

## Conclusion

Over the past three decades, IFITMs have been considered as intrinsic cellular factors that restrict a broad range of viruses. IFITM proteins restrict viruses at three distinct stages of the viral life cycle: blocking viral entry by trapping virions in endosomal vesicles; inhibiting viral gene expression and viral protein synthesis; incorporating into virions during viral assembly and subsequently reducing the infectivity of viruses. However, the precise mechanism underlying their functions remains to be further determined. More investigative works are still required to define the antiviral properties of IFITMs and how viruses escape from IFITM restriction. With respect to treatment, IFNs are commonly used medication for various diseases, such as chronic myelogenous leukemia (CML) (Preudhomme et al., 2010), HIV-associated Kaposi sarcoma (KS) (Gill et al., 1999), and HCV infection (Waziry et al., 2017), and their efficacy against viral diseases is achieved by the expression of ISGs including IFITMs. Unfortunately, IFN therapy can cause constitutional, neuropsychiatric, hepatic, and hematologic complications (Jonasch and Haluska, 2001). Recent studies have demonstrated that gp130, a transmembrane receptor, is also involved in regulation of IFITM expression (Bailey et al., 2012), suggesting that the gp130 agonist could be used in the treatment of viral diseases by inducing IFITMs and might avert the side effects of IFNs via bypassing IFN-regulated signaling. Moreover, due to the effect of IFITMs on lentiviral vector transduction as described earlier, it may provide a novel idea for gene transduction as well as disease treatment. However, further studies are still needed to better understand their application.

## Author Contributions

YL performed the systematic literature review and wrote the manuscript. MG, XY, BZ, and S-HC edited the manuscript. J-LC organized and provided the frame for the manuscript and critically revised the manuscript. All authors read and approved the final manuscript.

## Conflict of Interest Statement

The authors declare that the research was conducted in the absence of any commercial or financial relationships that could be construed as a potential conflict of interest.
